# ACcoding: A graph-based dataset for online judge programming

**DOI:** 10.1038/s41597-024-03392-z

**Published:** 2024-05-29

**Authors:** Kairui Chen, Fuqun Huang, Zejing Liu, Haomiao Yu, Liuchang Meng, Shasha Mo, Li Zhang, You Song

**Affiliations:** 1 Beihang University, School of Software, Beijing, 100191 China; 2 Western Washington University, Department of Computer Science, Bellingham, 98225 USA; 3 Beihang University, School of Cyber Science and Technology, Beijing, 100191 China; 4 Beihang University, School of Computer Science and Engineering, Beijing, 100191 China

**Keywords:** Education, Electrical and electronic engineering

## Abstract

A well-designed educational programming dataset is a valuable asset for students and educators. Such a dataset enables students to improve their programming performances continuously, provides researchers with significant data sources to identify students’ learning behaviours and enhance the quality of programming education. Several existing datasets for programming education are either limited by a small number of participating students or a short span of learning records, bringing great challenges to investigate students’ learning patterns in programming. We present a graph-based large-scale dataset specialized in programming learning on Online Judge (OJ) platform. The dataset, named *ACcoding*, was built by a university teaching group. As of the submission date of the initial manuscript of this paper (May 6, 2022), the dataset contains 4,046,652 task-solving records submitted by 27,444 students on 4,559 programming tasks over a span of 6 years. The large size of the dataset, combined with rich functional features, empowers educators to trace students’ programming progress and choose appropriate programming tasks for specific training purposes. We also presents examples of applications used by the dataset.

## Background & Summary

Online learning systems^1^ and intelligent tutoring systems^2^ have progressed significantly in recent years. Higher educational institutions started incorporating online learning systems into courses. To better understand students’ learning behaviours, researchers use various data mining technologies to analyse heterogeneous educational data collected from different online learning systems, leading to the emerging area of Educational Data Mining (EDM).

Educational data mining involves various tasks, including tracing the knowledge state of a student^3^, providing personalised feedback^4^, and making recommendations on learning activities^5^. The existing EDM studies focus on educational datasets of Math (ASSITments), English (EdNet), and Physics (USNA Physics), and limited studies have been found on datasets of computer programming^6^.

The existing studies on EDM of programming are similar to that of Math, English and Physics, such as improving the adaptive learning capabilities of online learning platforms by mining student interaction data, predicting students’ programming behaviours and analysing the error messages of source codes^7–9^. However, the education scenario of computer programming is significantly different from the aforementioned subjects in the following aspects: (1) The answer to a question is not simple options (e.g. A, B, C, D), but source codes in the text format; (2) A question may have multiple correct answers (source codes) and these source codes could be completely different; (3) An online programming platform often has various feedback types beyond simple correctness, including the running state of the code, such as memory and time overhead incurred during execution; (4) Repeated answering is often restricted or ignored in traditional scenarios, but questions can be answered repeatedly in online programming platforms. Therefore, the programming scenario is more complicated than traditional educational scenarios, which brings great challenges to analysing the programming learning behaviours of different students.

Another limitation of existing programming datasets is a small number of tasks or a short span of records. This limitation makes it hard to reveal students’ learning patterns. For instance, the Hour of Code dataset^10^ has only two tasks. The Blackbox dataset^11^ boasts over one hundred thousand users. However, the types of data collected are restricted to information that can be gathered through Integrated Development Environment (IDE) tools. This includes activities such as editing, compiling, execution, and instantiation of objects. The Code4Bench^12^ dataset lacks task labels, and focuses more on the area of code analysis than on computer programming education. The dataset presented in this paper, *ACcoding*, has overcome these limitations. It contains 4,046,652 programs submitted by 27,444 students for 4,559 programming tasks over a span of 6 years. Each task is tagged with a 10-point scale difficulty level and a coverage rating of skill sets on a 100-point scale. The dataset was collected through programming contests and the students’ daily exercises. In addition, the *ACcoding* dataset is constructed as dynamic programming knowledge graphs. With this graphic representation feature, a user’s submission history and the feedback he/she has received can be extracted.

Overall, the main advantages of the proposed dataset are: (1) This dataset is large-scale and versatile, targeted at improving programming studies of university students. (2) This dataset supports various EDM tasks, including knowledge tracing, learning path recommendation and error messages analysis. (3) This dataset encompasses extensive student records across various grades and programming proficiency levels. It boasts the highest number of submissions among existing programming datasets. (4) This dataset has multiple feedback types for submissions, making it unique among educational datasets and bringing challenges for knowledge tracing tasks. (5) To the best of our knowledge, it is the first dataset to describe programming learning entirely in knowledge graph structures, which offers unprecedented intelligibility and interpretability for EDM research.

The comparisons between *ACcoding* and existing educational datasets are summarized in Table 1.

**Table 1:** Comparison between *ACcoding* and existing educational datasets.

Datasets	ACcoding	Hour of Code		Code4Bench	CodeChef	ASSISTments		Junyi Academy
		Hoc4	Hoc18			2009	2012	
#users	27,444	509,405	263,569	57,775	61,245	4,417	46,674	247606
#tasks	4,559	1	1	2,990	1,474	26,688	179,999	722
#submissions	4,046,652	1,138,506	1,263,360	3,421,357	935,543	346,860	6,123,270	25,925,922
#submissions per student	**147.45**	2.23	4.79	58.22	15.28	78.53	131.19	104.71
#contests	**606**	—	—	541	—	—	—	—
#tags	100	—	—	—	—	124	266	41
Subject	Programming	Programming	Programming	Programming	Math	Math		
User Group	**University students**	K-8 students	Programming contestants	Programming contestants	Grade 4–12 students	K-8 students		
Time Range	2015.10-2022.5	2013.12-2014.3	2010.5-2017.8	—	2009–2010	2012-2013	2012.10-2015.1	

### The framework of *ACcoding*

An online judge (OJ) is an online platform that provides programming tasks for users to solve, and ideally may provide feedback to submissions in time. Most existing OJ platforms are designed for programming contestants (e.g. CodeForces) or job seekers (e.g. LeetCode^13^). These OJs are not targeted at early learners in computer programming. Therefore, such platforms do not offer functions for continuously improving users’ programming performances, such as tracking the programming learners’ learning paths, nor supporting computer education in higher education institutions. In contrast, *ACcoding* is an online interactive programming learning platform that focuses on the needs of college students, aiming to improve students’ programming ability and assist professors’ teaching, through analyzing students’ learning behaviors and regularly evaluating their programming performances. This is a public platform where any student and teacher can register.

**Fig. 1:**
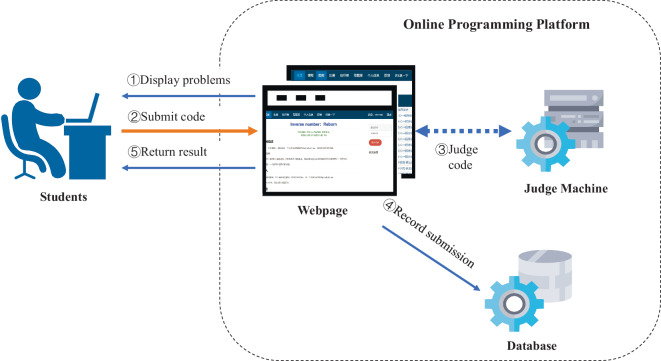
The framework of the *ACcoding* platform.

The running process of the *ACcoding* is illustrated in Fig. 1. First, the *ACcoding* displays a programming task to users through a web page, a user reads the task description, works out a solution and submit the source code. In the second phase, a judge machine compiles and executes the source code to determine whether the program passes all test cases, and whether it satisfies the predefined time and memory limit. Finally, the feedback of the program is returned to the user almost instantly, and the submission information is recorded in the *ACcoding* database.

Like most OJs, *ACcoding* provides two types of usage scenarios: daily exercises and programming contests. In an everyday exercise scenario, students are allowed to freely choose tasks in the task base and submit their solutions at any time. For the programming contests, a set of well-designed tasks is displayed to students; the students can choose the tasks from the set and try to solve as many tasks as possible within a limited time. In addition to the instant feedback for each submission, the students also receive their rankings of the contest. The ranking is calculated based on the number of correctly solved tasks and the total time of all the submissions.

## Methods

### Raw data

The main part of *ACcoding* is raw submission logs of daily and contest exercises. The submission logs elaborately record the programming learning behaviors of 27,444 undergraduates for about six years. The raw log records are organized in chronological order, as shown in Table 2.

**Table 2:** A sample of the raw data.

id	lang	result	score	t_cost	m_cost	length	detail	created_at	creator	task	contest	judge
…	…	…	…	…	…	…	…	…	…	…	…	…
499270	c	AC	1	8	1496	622	Accepted | 1 * (1/7) |...	2017/12/15 19:23	14824	1070	NA	3
499271	c++	PE	0.2	8	1512	254	Presentation Error | 0 *...	2017/12/15 19:23	14815	925	NA	-
499272	c	PE	0.2	8	1512	254	Presentation Error | 0 *...	2017/12/15 19:23	14815	925	NA	4
499273	c	WA	0	0	1428	422	...	2017/12/15 19:24	15944	1076	191	4
499274	c	AC	1	6	1504	256	Accepted | 1 * (1/10) |...	2017/12/15 19:24	14815	925	NA	4
499275	c++	AC	1	3	2680	319	Accepted | 1 * (1/3) | 1...	2017/12/15 19:25	14711	1069	NA	3
499276	c	AC	1	3	1436	814	Accepted | 1 * (1/3) | 1...	2017/12/15 19:25	15209	840	NA	3
…	…	…	…	…	…	…	…	…	…	…	…	…

Except for an extra “contest_id” field in the contest exercise records, other fields remain identical for contest and daily exercise records, including the source code information (programming language, submission time, time cost and memory cost, etc.), the feedback information (feedback type, detail, score) and the identification information (user id, task id, judge id). Specifically, *lang*, *t_cost* and *m_cost* are short for language, time cost (ms) and memory cost (KB), respectively. The variable *created_at* indicates the time of submission and *creator* indicates the id of the student who created this submission. Similarly, *task*, *contest*, *judge* denote the corresponding IDs. “NA” in the *contest* column means the corresponding task belongs to daily exercises. *ACcoding* provides multiple types of feedback to the users, as described below:

- Accepted (AC): The output of the program is the same as the standard answer, which means the program is correct.
- Wrong Answer (WA): The output of the program is incorrect.
- Time Limit Exceed (TLE): The program runs for longer than the specified maximum time.
- Memory Limit Exceed (MLE): The memory required for running the program exceeds the specified limit.
- Runtime Error (RE): The program performs an illegal operation, resulting in failure. Division by zero and out-of-bounds are examples of run-time failures.
- Presentation Error (PE): The data output by the program is correct, but the format does not conform to the requirements.
- Compile Error (CE): Source code fails during compiling.
- Other Error (OE): Errors that cannot be classified into any of the above categories.

### Data extraction and process

To build a comprehensive dataset for computer programming education, we collected raw submission logs of daily and contest exercises from the *ACcoding*. The collection and use of data are explained at the user registration stage, details of *ACcoding* service terms can be found at the bottom of website^14^, where we point out and emphasize that we will only collect information such as codes and submission records without involving any personal information related data, such as email address, student ID, school, and ultimately the user’s consent is obtained. These daily or competition exercises are drawn from a range of courses covering introductory to advanced computer programming, such as C programming, data structures, and algorithm analysis. The exercises encompassed in this set were created by educators or teaching assistants responsible for instructing the respective courses. These exercises, as well as the students who participated, effectively span the various phases of the programming learning journey, ranging from introductory levels for juniors to advanced levels for seniors.

**Fig. 2:**
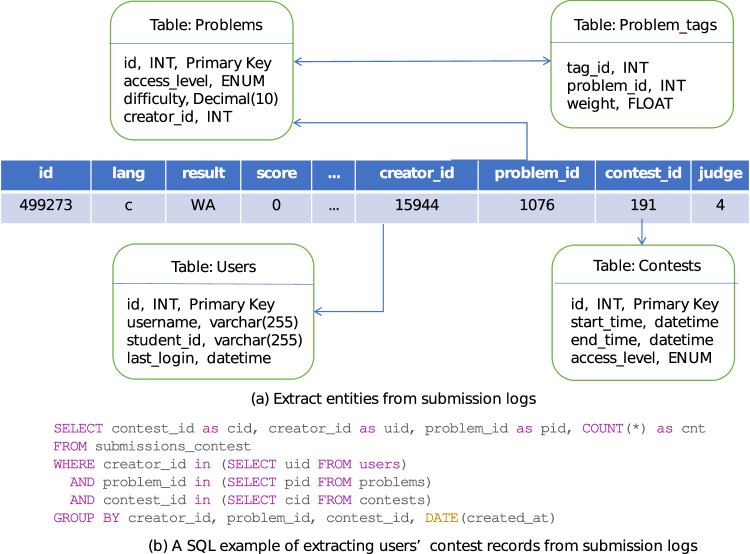
(**a**) Extract information of entities that appeared in submission logs. (**b**) A SQL example that extracts users’ number of submissions in contests from submission logs.

Logs of submissions come from 27,444 undergraduates for about 6 years of programming study. A sample of the submission log is shown in Table 2. Based on these submission logs, we collected information of entities that appeared in the submission logs, including user attributes, task and contest entities, and relations between these entities. As depicted in Fig. 2(a), we associate one submission record with its corresponding table based on the information provided in fields such as problem ID, contest ID. This association allows for the extraction of additional information, including the difficulty of the corresponding problem and knowledge points, as defined below:

- Difficulty level: the extent of how difficult to solve the problem. Difficulty level is measured in 10-point scale, where 1 indicates very easy while 10 indicates very difficult.
- Knowledge point: an area of knowledge that is essential for solving the problem. Each knowledge point is assigned a tag, such as sorting, iteration, greedy algorithm.

By traversing through all the submission records, the complete contents of the corresponding user table, topic table, and other related tables can be obtained. It is essential to emphasize that we did not gather the precise content of all the tasks, as only a subset of questions is publicly accessible to all users. We believe that the available information regarding difficulty levels, tags, and related details is adequate for the purposes of EDM research.

In the process of data extraction and processing from *ACcoding*, we first remove users’ personal information: The data we collect does not contain any important personal information such as phone numbers, addresses, etc. Users’ passwords, student ID numbers, and other information are encrypted by SHA-1 algorithm so that even the developers cannot know its real content when they view the database. After skipping these fields, we obtain users’ activity and submission results from logs by SQL. An example of writing SQL statements to extract users’ contest records is shown in Fig. 2(b).

Furthermore, when corresponding entities are extracted from the submission logs, tags of programming tasks are also extracted, even though they do not directly appear in logs. This is because these tags, which are annotated by experts, reflect knowledge points of programming tasks and help to conduct EDM tasks, such as knowledge tracing. Knowledge points (KP) refer to the knowledge of algorithms included or involved in a programming task, e.g., quick sort, finding the shortest path.

### The graphic representation

**Fig. 3:**
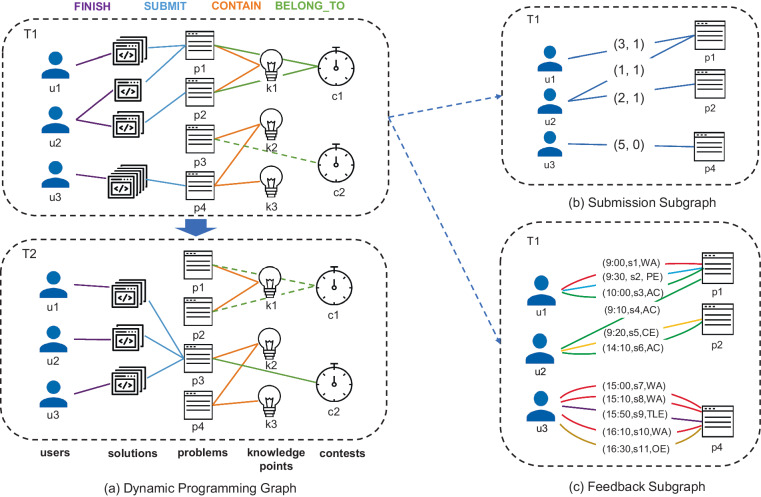
Contents and relationships of *ACcoding* knowledge graphs and subgraphs.

Since the *ACcoding* dataset has multiple types of entities and relations, it is intuitive to organize the *ACcoding* dataset into a knowledge graph structure, as illustrated in Fig. 3. As students submit task solutions dynamically, the programming graph pertains to be a dynamic knowledge graph. Figure 3(a) shows that the entity-to-entity relations are shown by lines. In particular, the solid line for BELONG TO relation means that the contest is currently in progress, while the dotted line indicates the contest has ended or has not started yet. The graph structure shown in Fig. 3(b) is a subgraph extracted from a programming graph in (a) based on the submission behavior. The repetitive submissions are aggregated into a single edge with a tuple (repeat count, correct or incorrect) as its attribute. Figure 3(c) shows another graph structure, a subgraph extracted from a programming graph in Fig. 3(a) based on the feedback results. The edge attribute in (c) is a Tuple with three elements, that is (time, solution id, feedback result).

A Submission Subgraph, as illustrated in Fig. 3(b), can be regarded as a lightweight graph that simplifies the details of repetitive submissions and ignores the diversity of feedback result types. It resembles traditional education datasets in terms of the content, and therefore, the Submission Subgraph can be used for generic EDM applications. On one hand, the Submission Subgraph can be used to infer the task preference of a student, and then provide task recommendations. On the other hand, the sequence of task-answer pair can be easily extracted from multiple Submission Subgraphs, which are the standard input data formats for general knowledge tracing models.

The submission graph provides a concise summary of students’ submission activities, while more fine-grained information are provided by the Feedback Subgraph, shown in Fig. 3(c).

A Feedback Subgraph is a knowledge graph with multiple edges, as one student can submit several solutions for a task, while different submissions could get different feedback types. The Feedback Subgraph makes the knowledge tracing tasks more challenging because various feedback types are introduced. Meanwhile, the detailed feedback information would be helpful for knowledge tracing models to depict the potential knowledge state of a student.

The knowledge graph structure reflects potential non-Euclidean structure in the *ACcoding* dataset, thus making it possible to utilize knowledge graph representation. From the application perspective, incorporating graphic structures into the knowledge tracing model as a relational inductive bias can improve performance, and enable a seamless integration of the corresponding knowledge into EDM applications.

## Data Records

### Dataset description and storage

The latest release of *ACcoding* dataset^15^ is available at https://zenodo.org/record/6522395, with 10.5281/zenodo.6522395, under a Creative Commons Attribution 4.0 International license. Entities are extracted and stored in a relational database in the form of tables. Note that in Table 3, names in lower cases are entities, and those in upper cases are relations between the entities. The programming graphs and subgraphs in Fig. 3 can be obtained from these tables, e.g., user-task information in the Feedback subgraph can be obtained from the Solutions table; similarly, a SQL statement like select-count-group_by can be used to obtain the user-submission records needed for the Submission subgraph.

**Table 3:** Content of collected data.

Name	Description	Attributes	Quantity
Users	undergraduate users	id	27,444
Problems	programming tasks	difficulty, time_limit, memory_limit	4,559
Contests	regular ACM contests	start_time, end_time	606
Problem-tags	knowledge point (KP) tags of tasks	tag_id, problem_id, weight	100
Submissions	source code submitted by users	code, language, time_cost, memory_cost	4,046,652
FINISH	links between users and submissions	—	4,046,652
SUBMIT	links between submissions and tasks	time, result	4,046,652
CONTAIN	links between tasks and KPs	—	4,397
BELONG TO	links between contests and tasks	order	4,756

**Fig. 4:**
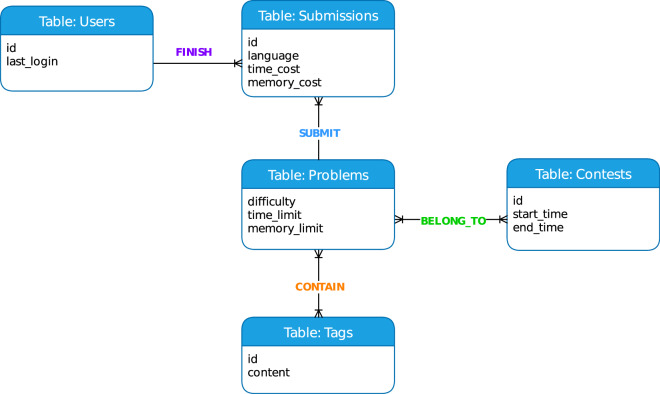
Entity-relation graph in *ACcoding* dataset.

The final tables in dataset are described in Fig. 4. The relationships between users-submissions, problems-submissions are one-to-many relations, while the relationships between problems-contests, problems-tags are many-to-many relations. To explore the problems-contests relationship, one can query the submissions table to identify submission records sharing the same problem ID across multiple distinct contest IDs. This scenario illustrates that identical problem may feature in different contests, while each contest may encompass various problems, thereby establishing a many-to-many relationship.

### Dataset statistics

**Fig. 5:**
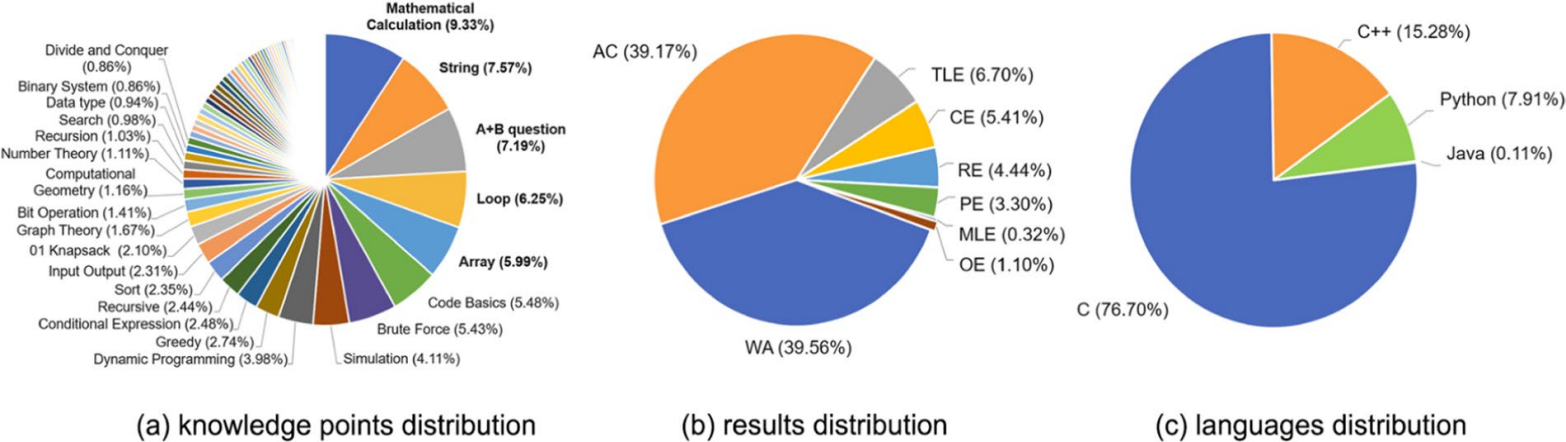
Statistics of the *ACcoding* dataset.

Figure 5 shows the statistics of the *ACcoding* dataset. Figure 5(a–c) are the distribution of knowledge points, feedback result types and submission languages, respectively.

The *ACcoding* dataset contains 100 tags for knowledge points collated by professors and teaching assistants. Figure 5(a) illustrates the percentage of tasks with different knowledge point tags. *ACcoding* covers various fundamental knowledge points such as array and conditional expression, and includes advanced knowledge points as well, e.g. dynamic programming and computational geometry. Tasks with the top 5 knowledge points are basic programming tasks, with a total proportion of 36.33%.

Figure 5(b) illustrates the proportion of submissions with different feedback types. We observe that AC submissions account for about 1/3 of the total submissions. Among all feedback types, WA submissions have the largest proportion, indicating that these submitted solutions commonly involve some elusive bugs and couldn’t pass all test cases. Furthermore, TLE, RE, PE and CE are relatively evenly distributed, while the remaining feedback types are rare in the *ACcoding* dataset. We also note that all solutions are written in C, C++, Python and Java programming languages, in which C and C++ submissions holds 91.98% of all submission, as shown in Fig. 5(c).

## Technical Validation

In this section, we conduct several EDM tasks on the *ACcoding* dataset, including programming knowledge tracing and task recommendation, which demonstrate the reliability and better performance of analyzing students’ learning behavior in computer programming study. Furthermore, we illustrate how to combine the *ACcoding* knowledge graph structures with relevant EDM applications.

### Tracing the programming knowledge students gained

#### Task and models

Knowledge tracing aims to model students’ knowledge state based on their learning histories and predict their performance for future interactions. In this task, several widespread knowledge tracing models are evaluated on the *ACcoding* dataset, including Deep Knowledge Tracing (DKT), Dynamic Key-Value Memory Networks (DKVMN)^16^ and Graph-based Knowledge Tracing(GKT). DKT leverages a single hidden vector modeled by a recurrent neural network (RNN) to represent the knowledge state. DKVMN utilizes memory augmented neural networks (MANNs)^17,18^ to model the learning process by two memory matrices: one is a static matrix for storing concepts, the other is a dynamic matrix for storing and updating knowledge states. GKT considers the complex graph structure of concepts and reformulates the knowledge tracing task as a time-series node-level classification task in the GNN. To construct the implicit concept graph of a dataset, GKT utilizes statistics approaches, including Dense graph, Transition graph and learning-based approaches, including Parametric adjacency matrix (PAM), Multi-head Attention (MHA)^19^, Variational autoencoder (VAE)^20^ based graphs.

#### Cross-dataset validations

We chose both the *ACcoding* dataset and ASSISTment2009, a popular benchmark math dataset, to evaluate the performance of all compared knowledge tracing models. For fairness, we only utilized the daily exercise data of the Submission Subgraph in 2019 for comparison, namely OJ2019, since the submission data in 2019 contains 131,612 interaction logs of 3650 students, which achieves the highest data density among all years. We organized the knowledge tracing data of both datasets as answer sequences with knowledge-point and answer result pairs (*k*_*t*_, *a*_*t*_) for each student, where *k*_*t*_ is the knowledge point and *a*_*t*_ ∈ {0, 1}. Hence, traditional knowledge tracing can be regarded as a binary classification task. The experiments also extended the traditional knowledge tracing task as multiple classification tasks for the programming education scenario. As discussed in Section Data Records, an OJ platform often contains multiple feedback types, such as AC, WA, TLE, etc. These feedback types were chosen prediction targets in the multi-class knowledge tracing task of the OJ2019 dataset.

#### Parameters and training

For the ASSISTment2009 dataset, the settings and hyper-parameters were kept the same as shown in the GKT paper for the best performance. For the *ACcoding* dataset, we set the dimension of hidden layers as 200, 32, 32, and the learning rate of Adam^21^ optimizers as 0.001, 0.01, 0.001 for DKT, DKVMN and GKT models, respectively. In this case, OJ2019-binary dataset use 0–1 targets, where 1 means AC feedback type, and 0 means other types. OJ2019-full dataset use all feedback types as prediction targets. The batch size of all knowledge tracing models was 128, and both datasets were divided into training, validation and test sets with the proportion of training: validation: test = 6:2:2.

#### Results analysis

We utilized the area under the curve (AUC) metric to evaluate the performance of all compared knowledge tracing models, as shown in Table 4. We observed that all knowledge tracing models achieve better performances on the *ACcoding* dataset, even though its train set was smaller than that of ASSIST2009, which implied the intrinsic difference among different subject datasets. Furthermore, the AUC values of all compared knowledge tracing models decreased on the OJ2019-full dataset compared to the OJ2019-binary dataset. It suggests that predicting feedback types for the programming submissions is more challenging than predicting for a binary variable that includes either correct or incorrect status. We also see that GKT variants achieved the best performance across all three knowledge tracing methods and DKVMN performance is better than the DKT model. It demonstrates that modeling the implicit graph structure of knowledge states in GKT is more effective than only modeling knowledge states by a single hidden vector in DKT and several memory vectors in DKVMN. Note that the performances of all GKT variants were similar in these datasets. This indicates that statistical graph is a simple but effective way of modeling implicit concept relations in the GKT model.

**Table 4:** The benchmark performance of programming knowledge tracing tasks on both ASSIST2009 and the proposed dataset.

KT Model		AUC		
		ASSIST2009	OJ2019-binary	OJ2019-full
DKT		0.709	0.714	0.709
DKVMN		0.710	0.738	0.717
GKT	Dense	0.722	0.744	0.727
	Transition	0.721	0.749	0.735
	PAM	0.719	0.748	0.728
	MHA	0.723	0.753	0.722
	VAE	0.722	0.741	0.719

### Programming task recommendation for personalized learning

#### Task and models

Due to the knowledge graph structure of our dataset, it is interesting to explore intelligent task recommendation. We chose BiNE (Bipartite Network Embedding Ming)^22^, IGE (Interaction Graph Embedding)^23^ and RHINE (Relation Structure-Aware Heterogeneous Information Network Embedding)^24^, R-GCN (Relational Graph Convolutional Networks)^25^, and our modified version of the Feedback Subgraph based on R-GCN (called LSTM-RGCN) to learn the student and task representations in our dataset, and thus validate the generalizability and tractability of *ACcoding* dataset for educational recommendation tasks. We selected these models because they concern heterogeneous networks, interactions, relations and adaptive neighborhood sequences for nodes, respectively; these are also the characteristics of our graph. In addition, three classical knowledge graph embedding models TransE^26^, TransH^27^, TransR^28^ were used for reference since they are also applicable to describe the overall structure of the programming graph.

#### Data generation

Data were extracted from the Submission and Feedback Subgraphs, and the differences were reflected in the edges. The data for BiNE came from the Submission Subgraph, with the weight of the edges being the number of submissions, without feedback results. IGE used a Feedback Subgraph with each edge having a date (the time of submission) and an attribute (a list of results for users submitting the same task multiple times). According to the sparsity measure proposed in RHINE, we captured the user-task relation as Interaction Relations (IRs). In contrast, task-knowledge point and user-task-knowledge point as Affiliation Relations (ARs), with a single-sided weight of 1 and the relational chain weights were calculated. Knowledge graph embedding models used a Feedback Subgraph with only relational type attributes on the edges, including, contain/unique (task and knowledge are one-to-one) contain/multiple (one-to-many) and submit/daily/result(feedback results). The R-GCN data were extracted from the Submission Subgraph with the final AC as the relationship type and the number of repeats as the weight. LSTM-RGCN used a Feedback Subgraph with the feedback results type and time to mark the dynamic process.

#### Parameters and training

The classical recommended assessment metrics F1-Score, Mean Reciprocal Rank (MRR) and Normalized Discounted Cumulative Gain (NDCG) score were used for the final assessment of the performance of recommendation on encoder models. We chronologically sampled 70% as the training set and the remaining 30% as the testing set. The last 40% of the training set was used as the validation set. The validation set was used to evaluate the models during the iterative process (in terms of MRR), and the best-performed model was selected for testing. For each method’s recommendation experiment, we computed the inner product of the student and task feature vectors for feature interaction. Then, the student’s preference for the task was predicted, and the Top-10 results were evaluated for performance.

#### Results analysis

The performance of graph embedding models in the recommendation task is shown in Table 5. RHINE outperforms the other methods, for it distinguishes between interaction and subordination with a larger granularity. It improves model identification compared to BiNE and IGE, which only considers interaction relationships. It avoids overfitting compared to the meticulous latter models that consider each feedback result. Our improved LSTM-RGCN is suboptimal and significantly better than the original RGCN, indicating that capturing each node’s neighborhood sequence and introducing personalized hidden embeddings can yield better results.

**Table 5:** Task recommendation performance of all compared methods.

	Method	F1@10	MRR@10	NDGG
Graph Embedding	Embedding	0.028	0.039	0.025
	IGE	0.012	0.057	0.017
	RHINE	0.041	0.088	0.043
Knowledge Graph Embedding	TransE	0.022	0.048	0.021
	TransH	0.032	0.089	0.034
	TransR	0.021	0.044	0.020
Graph Neural Network	RGCN	0.014	0.033	0.016
	LSTM-RGCN	0.036	0.063	0.033

In addition to helping the algorithm improve accuracy, the graphic structure of the *ACcoding* dataset allows the algorithm to focus on interpretability improvements. We tested the *ACcoding* dataset using typical interpretable algorithms RippleNet^29^ and CAML(Co-Attentive Multi-Task Learning for Explainable Recommendation)^30^. These algorithms made full use of the relational information in the knowledge graph and pooled the entity vectors of each layer in a weighted manner, and therefore, they can depict the user’s preferences in more detail and provide recommendation explanations to the user. Table 6 shows the Top 5 recommendation results and their corresponding recommendation explanations obtained from the interpretable algorithms.

**Table 6:** Examples of the top 5 recommendation results and their corresponding recommendation explanations.

Recommended Tasks	Recommendation Explanations
Repeat String Pairs	A classmate with similar interests to yours also did this programming task.
Tree Structure	Based on the programming task you did, “Mdd’s Chain Table”, we recommend this programming task with similar difficulty.
Minimum spanning tree algorithm	Based on your mastered knowledge point of the “KD tree”, we recommend this programming task with similar knowledge point.
Multipacks	Based on your mastered knowledge point of the “Knapsack problem”, we recommend this programming task with similar knowledge point.
Crowdsourcing Issues	A classmate with similar interests to yours also did this programming task.

### Programming graph analysis

There are current problems in online education. For instance, during the teaching process, sometimes teachers do not get timely feedback from students, students’ orientation and learning paths are not clear, teachers’ and students’ data management is cumbersome, etc. Applying the *ACcoding* dataset combined with knowledge graph tools can effectively solve these problems. To demonstrate the potential value embedded in the *ACcoding* knowledge graphs, three popular knowledge graph embedding methods—TransE, TransH, TransR—are used for node information representation and programming graph analysis. Since the primary function of *ACcoding* is to provide feedback to users for each task, the most critical nodes are users and tasks. The popularity and difficulty of tasks provide valuable information regarding the habits and abilities of users.

#### Analysis of task classification results

##### Task and model

**Fig. 6:**
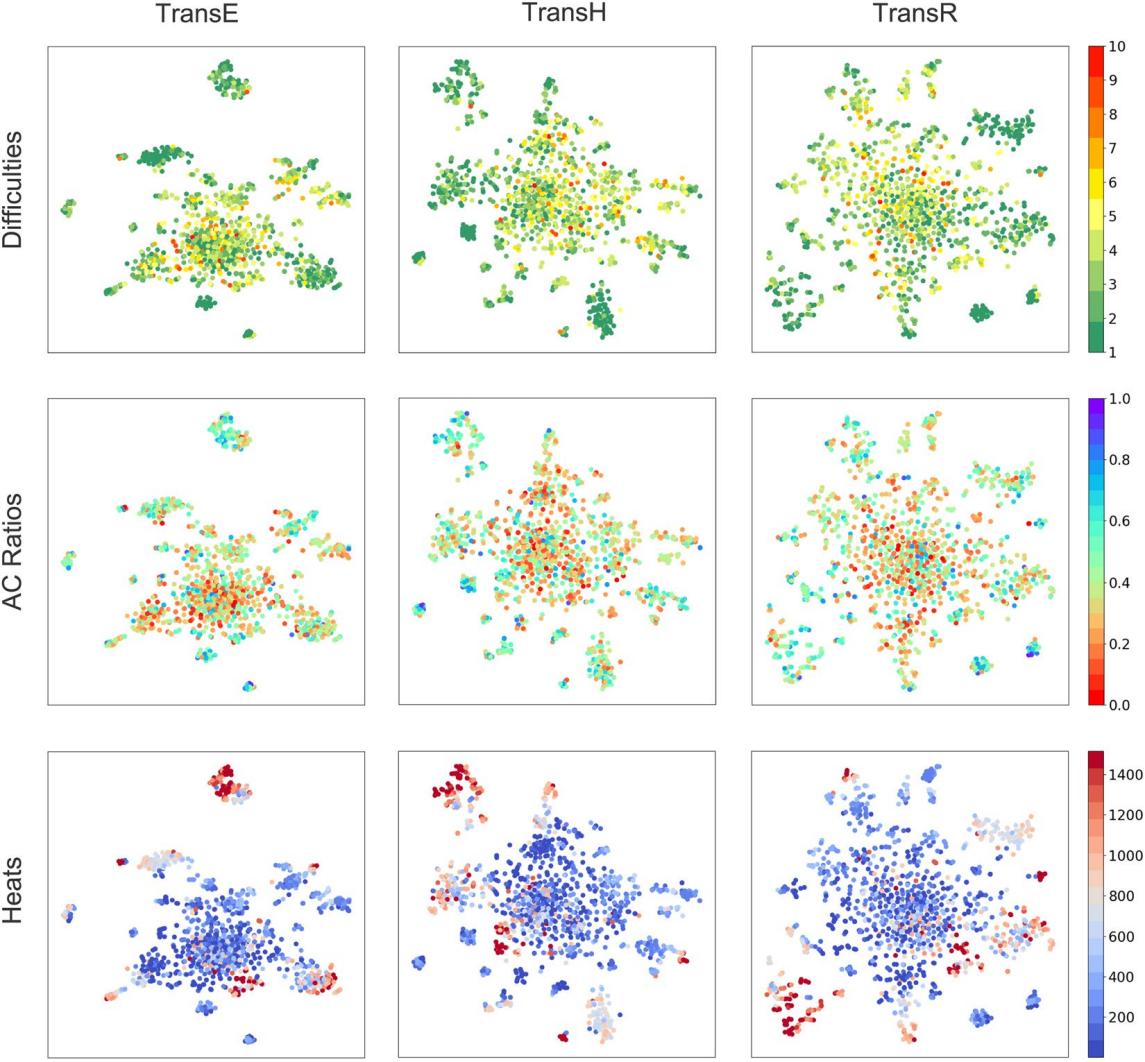
Visualization of task embeddings. Heats: The number of people who attempted the task. AC Ratio: The percentage of ACs for tasks of submissions.

To test whether the programming graph contains underlying crucial information about the task, we use t-SNE(t-Distributed Stochastic Neighbor Embedding)^31^ to map the learned task vectors into 2-dimensional scatter plots. Each task node is labeled with three dimensions: difficulty, AC ratio and heat, as shown in Fig. 6. The difficulty is assigned by an instructor when the task was created. Heat and AC Ratio are derived from the submission data,and their definitions are as follows:

- AC Ratio: The number of submissions that received the feedback type “Accept” (AC) for a task divided by the total number of attempted submissions for that task.
- Heat: The number of attempted submissions for a task.

##### Results analysis

Overall, the visualized scatter distribution of each dimension shows a clear trend from the edge to the center. Taking the results of TransE as an example, there are three dense clusters and several small clusters at the edges of the plots, which have low difficulties (below 3), high AC ratios (around 0.5), and high heat (about 1000). Though the main parts do not show clear boundaries, we find that the outer part is the transition between the edge and the center, while the tasks with great difficulty, low pass rate, and low heat tasks are concentrated in the centers. Heat is the most obvious among the three dimensions, which can be interpreted as the absolute quantitative dominance of the interaction edge in the knowledge graph. The results of TransH and TransR demonstrate similar distribution patterns.

Combining the three dimensions of the task reveals some patterns in student learning. The most popular and concentrated tasks have the highest accuracy rate but are not the easiest (with difficulties of 2 3). It means that tasks involving some simple algorithms are the most attractive for students and can be passed quickly. The cluster with the lowest difficulty corresponds to a medium degree of heat and accuracy, which is not reasonably as expected. An explanation is that these tasks tend to be selected by beginners who are unfamiliar with the programming or the environment. They make multiple attempts to get the correct answers, thus reducing the accuracy rate, meanwhile, after getting started, they do not try such simple tasks again, thus reducing the popularity of such tasks. There is also a hot and moderately complex cluster with an unfocused accuracy distribution at the edge of the center. These are the tasks including some classical algorithms with a considerable difficulty level, so there are many attempts with varied accuracies. In addition, the difficulty levels of other clusters are negatively related to both the AC ratios and the heat.

Looking horizontally at the dimensionality reduction results obtained by different methods, the scatter distribution is increasingly scattered from left to right. The simplest TransE method has the best aggregation, with three large clusters evident and dense in the middle, while the clusters of TransR are least aggregated but distinguished by color. This situation should be the complexity and the number of parameters of the three models. TransH and TransR introduce hyperplanes and relationship spaces to improve the discrimination of relationships. However, interaction relations account for an absolute majority of *ACcoding* data, so this distinction does not work, and homogeneous relations may reduce the differences between the points, making the distribution unfocused. Moreover, the projection introduces normal vectors and matrices, which require more parameters to be trained.

##### Remarks

As the visual analysis shows, the task vectors obtained by knowledge graph embedding models contain many features that are hard to observe directly, such as the relevance of the tasks and the choice preferences of users. This indicates the rich features provided by our dataset that can be further explored by interested researchers.

#### Analysis of user clustering results

##### Task and model

**Fig. 7:**
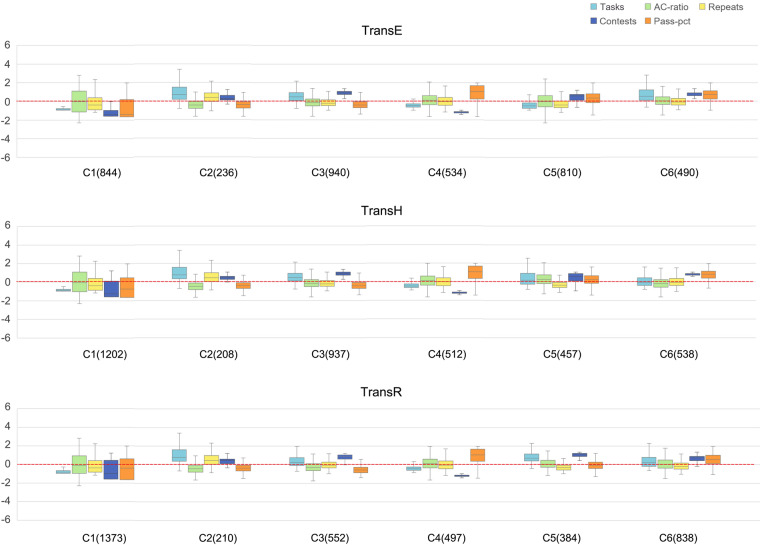
Clusters and features of user embeddings.

To analyze the user entities in the programming graph, we perform a k-means analysis for the user embedding, setting k = 6. The experiments showed that k=6 is the best performer, with significant differences between individual clusters and higher aggregation of features in all the same clusters. We analysed the performance of each cluster on five features, including Tasks, AC-ratio, Repeats, Contests, and Pass-pct. These features provide a comprehensive picture of the users in daily practice and time-limited competitions. Tasks and Contests reflect users’ activation in learning, AC-ratio and Pass-pct reflect programming ability, and combined Repeats reflect users’ learning habits. The values are z-score normalized so that they are distributed around 0. The results obtained by different methods are clustered into similar clusters, as shown in Fig. 7. In this figure, the first three features are daily practice characteristics. Tasks indicate the number of tasks submitted by a user. Repeats indicate how many times a user submits each task on average. The last two features are contest characteristics. Contests indicate how many contests a user has entered, and Pass-pct indicates the average pass rate of the user in the contest, calculated as the number of AC tasks/total number of contest tasks.

##### Results analysis

Intuitively, the C1 C4 features obtained by all three methods are consistent and differ only in quantity. Cluster C1 consists of the least active student who submit few tasks, participate in occasional contests, and have a low accuracy rate. C2 contains a minority of students who heavily rely on the feedback from the system: repeatedly submitting and fixing bugs. They attempt many tasks with low AC-ratio and high Repeats and some contests with low passing rates. C3 includes students with low level of persistence in learning. They submit many tasks with a low AC-ratio, while they often enter contests but have low passing rates. Students in the C4 group also practice less, but their AC-ratio and Repeats are more concentrated than that of C1. Although their Contests are low, their Pass-pct are the highest. This information is not directly observable in data but is explored through detailed information mining. The other two clusters yielded less identical results across methods, but both had high Contests and Pass-pct.

In addition, there are two particular groups, C4 and C5 of TransE and C3 and C5 of TransH. They do not show significant differences in daily features, but show substantial differences in the time-limited contest features. Considering the embedding vectors contain potential information about the students’ performance in their daily routine, these two particular groups indicate that the knowledge graph embedding has effectively captured the potential information. Also, it suggests that embedding is a more accurate description of the user, compared to the results obtained from the three daily performance features alone, has a better ability to predict student performance in the contest.

##### Remarks

The analysis of the above user embedding results implies that the information contained in the *ACcoding* data is rich and that students’ learning behavior is well worth studying. Researchers can mine user behavior through various graph embedding methods to build user profiles, and apply them to research areas such as personalized learning path recommendations.

#### Implications

The graph analysis of the *ACcoding* dataset can has significantly met the teaching and learning needs in real courses given by the university. From a student perspective, there are individual differences in students’ learning effort, learning ability, interests, etc. Based on clustering algorithms to analyze students’ task submissions and various other information, we can better understand the characteristics of each student, and thus help them improve their programming performances. From the teacher’s perspective, the Graph analysis helps teachers analyze how students master different tasks, so as to determine students’ learning levels and abilities to adjust their teaching plans and provide personalized tutorials in a targeted manner.

## Usage Notes

We have demonstrated (in the above section) how to use the *ACcoding* dataset to perform various EDM tasks, such as knowledge tracing, task recommendation, task classification and student clustering. There are some other exciting research directions as yet to be explored by using this dataset.

- **Student Performance Prediction**. The *ACcoding* dataset contains two types of data: daily exercise data and contest exercise data. Most contests are weekly contests of programming courses at university and the contest performance is a part of the final score in a specific programming course. Hence, we can use the daily exercise data to monitor the actual learning state of students and make an early prediction of their contest performances, this is helpful for lectures to master the class-agnostic student performance and adjust teaching plans flexibly^32^.
- **Learning Path Recommendation**. As shown in Table 1, the average interaction number of the *ACcoding* is the largest among all compared datasets, which provides the possibility for personalized recommendation. Furthermore, *ACcoding* has dynamic interaction records across an extended period. We can build personalized learning path recommendation applications on the *ACcoding* dataset based on these features. Although we only benchmarked the performances of several static knowledge graph embedding methods, it will be an exciting direction to incorporate dynamic(temporal) knowledge graph embedding methods with programming tasks or learning path recommendation systems.
- **Code Message Analysis**. The *ACcoding* dataset not only has massive interaction records (over 4 millions) but also source codes written by different programming languages. Context information, including feedback types, time costs, memory costs, and intervals between submissions, can be combined with source codes to imply whether and how fast a student fixes code errors.
- **Intelligent Programming Feedback**. Based on actual code message analysis results, we can move further to provide intelligent programming feedback for students. For instance, we can build a code error detection application that intelligently instructs students to find bugs in their WA solutions, which is especially helpful for programming beginners.

### Supplementary information

Supplymentary file

## Data Availability

All the code is freely accessible in https://github.com/KarryBramley/ACcoding-Dataset.

## References

[1] Thakkar, S. R. & Joshi, H. D. E-learning systems: a review. In *2015 IEEE Seventh International Conference on Technology for Education (T4E)*, 37–40 (IEEE, 2015).

[2] Woolf, B. P. *Building intelligent interactive tutors: Student-centered strategies for revolutionizing e-learning* (Morgan Kaufmann, 2010).

[3] Piech, C. *et al*. Deep knowledge tracing. In *Advances in neural information processing systems*, 505–513 (2015).

[4] Piech, C. *et al*. Learning program embeddings to propagate feedback on student code. In *International conference on machine Learning*, 1093–1102 (PMLR, 2015).

[5] Lan, A. S. & Baraniuk, R. G. A contextual bandits framework for personalized learning action selection. In *EDM*, 424–429 (2016).

[6] Ihantola, P. *et al*. Educational data mining and learning analytics in programming: Literature review and case studies. In *Proceedings of the 2015 ITiCSE on Working Group Reports*, 41–63 (2015).

[7] Altadmri, A. & Brown, N. C. 37 million compilations: Investigating novice programming mistakes in large-scale student data. In *Proceedings of the 46th ACM Technical Symposium on Computer Science Education*, 522–527 (2015).

[8] Kohn, T. The error behind the message: Finding the cause of error messages in python. In *Proceedings of the 50th ACM Technical Symposium on Computer Science Education*, 524–530 (2019).

[9] Júnior, A. S., de Figueiredo, J. C. A. & Serey, D. Analyzing the impact of programming mistakes on students’ programming abilities. *Brazilian Symposium on Computers in Education (Simpósio Brasileiro de Informática na Educação-SBIE)* **30**, 369 (2019). 10.5753/cbie.sbie.2019.369

[10] Du, J., Wimmer, H. & Rada, R. hour of code”: Can it change students’ attitudes toward programming? *Journal of Information Technology Education: Innovations in Practice* **15**, 53 (2016).

[11] Brown, N. C. C., Kölling, M., McCall, D. & Utting, I. Blackbox: a large scale repository of novice programmers’ activity. In *Proceedings of the 45th ACM technical symposium on Computer science education*, 223–228 (2014).

[12] Majd, A., Vahidi-Asl, M., Khalilian, A., Baraani-Dastjerdi, A. & Zamani, B. Code4bench: A multidimensional benchmark of codeforces data for different program analysis techniques. *Journal of Computer Languages* **53**, 38–52 (2019). 10.1016/j.cola.2019.03.006

[13] LeetCode - The World’s Leading Online Programming Learning Platform. https://leetcode.com/ (2024).

[14] OJ4TH. https://accoding.buaa.edu.cn (2024).

[15] Liu, Z. Accoding-dataset: v1.0.0* Zenodo*10.5281/zenodo.6522395 (2022). 10.5281/zenodo.6522395

[16] Zhang, J., Shi, X., King, I. & Yeung, D.-Y. Dynamic key-value memory networks for knowledge tracing. In *Proceedings of the 26th international conference on World Wide Web*, 765–774 (2017).

[17] Graves, A., Wayne, G. & Danihelka, I. Neural turing machines. *arXiv preprint arXiv:1410.5401* (2014).

[18] Weston, J., Chopra, S. & Bordes, A. Memory networks. *Eprint Arxiv* (2014).

[19] Vaswani, A. *et al*. Attention is all you need. *Advances in neural information processing systems* **30** (2017).

[20] Kingma, D. P. & Welling, M. Auto-encoding variational bayes. In* Proc. of ICLR* (2014).

[21] Kingma, D. P. & Ba, J. Adam: A method for stochastic optimization. In *Proc. of ICLR* (2015).

[22] Gao, M., Chen, L., He, X. & Zhou, A. Bine: Bipartite network embedding. In *The 41st International ACM SIGIR Conference on Research & Development in Information Retrieval*, 715–724 (2018).

[23] Zhang, Y., Xiong, Y., Kong, X. & Zhu, Y. Learning node embeddings in interaction graphs. In *Proceedings of the 2017 ACM on Conference on Information and Knowledge Management*, 397–406 (2017).

[24] Lu, Y., Shi, C., Hu, L. & Liu, Z. Relation structure-aware heterogeneous information network embedding. *Proceedings of the AAAI Conference on Artificial Intelligence* **33**, 4456–4463 (2019). 10.1609/aaai.v33i01.33014456

[25] Schlichtkrull, M. *et al*. Modeling relational data with graph convolutional networks. In *European semantic web conference*, 593–607 (Springer, 2018).

[26] Bordes, A., Usunier, N., Garcia-Duran, A., Weston, J. & Yakhnenko, O. Translating embeddings for modeling multi-relational data. In *Neural Information Processing Systems (NIPS)*, 1–9 (2013).

[27] Wang, Z., Zhang, J., Feng, J. & Chen, Z. Knowledge graph embedding by translating on hyperplanes. In *Proceedings of the AAAI Conference on Artificial Intelligence*, vol. 28 (2014).

[28] Lin, Y., Liu, Z., Sun, M., Liu, Y. & Zhu, X. Learning entity and relation embeddings for knowledge graph completion. In *Twenty-ninth AAAI conference on artificial intelligence* (2015).

[29] Wang, H. *et al*. Ripplenet: Propagating user preferences on the knowledge graph for recommender systems. In *Proceedings of the 27th ACM international conference on information and knowledge management*, 417–426 (2018).

[30] Chen, Z. *et al*. Co-attentive multi-task learning for explainable recommendation. In *IJCAI*, 2137–2143 (2019).

[31] Van der Maaten, L. & Hinton, G. Visualizing data using t-sne. *Journal of machine learning research* **9** (2008).

[32] Riestra-Gonz, M., del Puerto Paule-Ruiz, M. & Ortin, F. Massive lms log data analysis for the early prediction of course-agnostic student performance. *Computers & Education* 104108 (2020).

