# Author Correction: ACcoding: A graph-based dataset for online judge programming

**DOI:** 10.1038/s41597-024-03693-3

**Published:** 2024-08-01

**Authors:** Kairui Chen, Fuqun Huang, Zejing Liu, Haomiao Yu, Liuchang Meng, Shasha Mo, Li Zhang, You Song

**Affiliations:** 1 Beihang University, School of Software, Beijing, 100191 China; 2 Western Washington University, Department of Computer Science, Bellingham, 98225 USA; 3 Beihang University, School of Cyber Science and Technology, Beijing, 100191 China; 4 Beihang University, School of Computer Science and Engineering, Beijing, 100191 China

**Keywords:** Education, Electrical and electronic engineering

Correction to: *Scientific Data* 10.1038/s41597-024-03392-z, published online 29 May 2024

In this article the funding from the ‘key Research and Development Program of Hebei Province (21310101D)’ was omitted. The original article has been corrected.

