# Supplementary material for: ACcoding: A graph-based dataset for online judge programming
Source: Sci Data. 2024 May 29;11:548. doi: 10.1038/s41597-024-03392-z (PMC11137024; doi:10.1038/s41597-024-03392-z)
Supplement: Supplementary file 1 — Supplymentary file [file 41597_2024_3392_MOESM1_ESM.pdf]

Supplymentary

Contents

Privacy Policy 2
Information we may collect 2
Information you provide 2
Information you generate when using our services 2
How we may use information 2
Contact Us 2
Ethic Approval 3

Privacy Policy

Information we may collect

In providing our services, we may collect, store and use the following information about you. If you do not provide the relevant information, you may not be able to register as a user or enjoy some of the services we offer, or achieve the intended results of the relevant services.

Information you provide

- Personal information (not including passwords) that you provide to us when you register for an account, for example, school, college, email address.

Information you generate when using our services

We may collect the following information when you use our services:

- Log information
    - Information may be automatically captured by the system through cookies or other means when you use our services, including: device or software information, (such as configuration information provided by your mobile device, web browser or other program used to access), your IP address, etc;
    - Information you searched for or viewed while using our services, such as search terms you use, the url addresses of the web pages you visit, and other information or content details you view or request while using our services.
- Data Information
    - Data that is submitted or automatically generated when you use our services, such as code or text submitted to solve a problem, records of participation in a competition, etc.

How we may use information

We may use the information we collect to provide services for the following purposes:

- Providing services to you;
- To ensure the security of the products and services we provide to you, for example: identity verification, customer service, security , archiving and backup;
- To help us carry out research based on data from the OJ platform, for example: recommendation system, human factors analysis, etc;
- Converting your data (not including personal information such as school, student number, email address, etc.) into a public dataset to help conduct research related to the OJ platform, e.g. recommendation system, human factors analysis, etc.
- To help us design new services and improve our existing services;
- Enabling us to better understand how you access and use our services so that we can tailor our response to your individual needs;
- Involving you in surveys about our platform.

Contact Us

If you have any questions or comments about this Privacy Policy, please contact us

at oj_feedback@163.com

Ethic Approval

北京航空航天大学软件学院

北京航空航天大学软件学院科技伦理批准函

项目名称	ACcoding: A graph-based dataset for online judge programming
申请类别	科技研究

在本项目（下文简称 ACcoding）研究过程中，本项目主要收集用户提交的代码以及题目、比赛等信息。为了确保用户数据的安全和隐私，我们特此申请研究伦理审查。

在 ACcoding 研究中，我们主要收集了以下类型的数据：

- 用户提交记录（如代码、反馈结果等）
- 课程题目与比赛
- 题目知识点标签

我们不收集、存储或使用任何关于用户的个人信息，如姓名、邮箱、电话号码等。数据处理过程中严格遵循数据保护和隐私保护的原则。我们于 https://accoding.buaa.edu.cn/ 公开了我们的数据使用政策，以确保用户对其数据的使用有明确的了解。所有用户在使用我们的 OJ 系统之前，必须同意并遵守这些条款。

本研究的数据收集和处理符合中国科技部《科技伦理审查办法（试行）》(2023）审查要求，确保用户数据的安全和隐私。我们承诺将严格遵循上述原则，并确保所有数据的合法、合理和透明使用。

申请人：陈开睿　　　　2024 年 4 月 10 日

院（系）审批意见	经审查与讨论，认为其研究设计合理，符合用户知情与伦理原则，予以批准。<br><br>负责人（签字）：　　2024 年 4 月 10 日
